# Vulnerability of Large Language Models to Prompt Injection When Providing Medical Advice

**DOI:** 10.1001/jamanetworkopen.2025.49963

**Published:** 2025-12-19

**Authors:** Ro Woon Lee, Tae Joon Jun, Jeong-Moo Lee, Soo Ick Cho, Hyung Jun Park, Jungyo Suh

**Affiliations:** 1 Artificial Intelligence Research Committee, GIGA Study, Incheon, Republic of Korea; 2 Department of Radiology, Inha University College of Medicine, Incheon, Republic of Korea; 3 Department of Information Medicine, University of Ulsan College of Medicine, Asan Medical Center, Seoul, Republic of Korea; 4 Department of Surgery, Division of Liver & Liver Transplantation, Ewha Womans University Seoul Hospital, Seoul, Republic of Korea; 5 InSkin Lab, Seoul, Republic of Korea; 6 Helpmedoc Inc, Seoul, Republic of Korea; 7 Department of Urology, University of Ulsan College of Medicine, Asan Medical Center, Seoul, Republic of Korea

## Abstract

**Importance:**

Large language models (LLMs) are increasingly integrated into health care applications; however, their vulnerability to prompt-injection attacks (ie, maliciously crafted inputs that manipulate an LLM’s behavior) capable of altering medical recommendations has not been systematically evaluated.

**Objective:**

To evaluate the susceptibility of commercial LLMs to prompt-injection attacks that may induce unsafe clinical advice and to validate man-in-the-middle, client-side injection as a realistic attack vector.

**Design, Setting, and Participants:**

This quality improvement study used a controlled simulation design and was conducted between January and October 2025 using standardized patient-LLM dialogues. The main experiment evaluated 3 lightweight models (GPT-4o-mini [LLM 1], Gemini-2.0-flash-lite [LLM 2], and Claude-3-haiku [LLM 3]) across 12 clinical scenarios in 4 categories under controlled conditions. The 12 clinical scenarios were stratified by harm level across 4 categories: supplement recommendations, opioid prescriptions, pregnancy contraindications, and central-nervous-system toxic effects. A proof-of-concept experiment tested 3 flagship models (GPT-5 [LLM 4], Gemini 2.5 Pro [LLM 5], and Claude 4.5 Sonnet [LLM 6]) using client-side injection in a high-risk pregnancy scenario.

**Exposures:**

Two prompt-injection strategies: (1) context-aware injection for moderate- and high-risk scenarios and (2) evidence-fabrication injection for extremely high-harm scenarios. Injections were programmatically inserted into user queries within a multiturn dialogue framework.

**Main Outcomes and Measures:**

The primary outcome was injection success at the primary decision turn. Secondary outcomes included persistence across dialogue turns and model-specific success rates by harm level.

**Results:**

Across 216 evaluations (108 injection vs 108 control), attacks achieved 94.4% (102 of 108 evaluations) success at turn 4 and persisted in 69.4% (75 of 108 evaluations) of follow-ups. LLM 1 and LLM 2 were completely susceptible (36 of 36 dialogues [100%] each), and LLM 3 remained vulnerable in 83.3% of dialogues (30 of 36 dialogues). Extremely high-harm scenarios including US Food and Drug Administration Category X pregnancy drugs (eg, thalidomide) succeeded in 91.7% of dialogues (33 of 36 dialogues). The proof-of-concept experiment demonstrated 100% vulnerability for LLM 4 and LLM 5 (5 of 5 dialogues each) and 80.0% (4 of 5 dialogues) for LLM 6.

**Conclusions and Relevance:**

In this quality improvement study using a controlled simulation, commercial LLMs demonstrated substantial vulnerability to prompt-injection attacks that could generate clinically dangerous recommendations; even flagship models with advanced safety mechanisms showed high susceptibility. These findings underscore the need for adversarial robustness testing, system-level safeguards, and regulatory oversight before clinical deployment.

## Key Points

**Question:**

Can commercial medical large language models (LLMs) be manipulated through prompt-injection attacks (ie, maliciously crafted inputs that manipulate an LLM’s behavior) to recommend unsafe or contraindicated treatments?

**Findings:**

In this quality improvement study using a controlled simulation of 216 patient-LLM dialogues, webhook-simulated prompt-injection attacks succeeded in 94.4% of trials and 91.7% of extremely high-harm scenarios, including US Food and Drug Administration Category X pregnancy drugs such as thalidomide.

**Meaning:**

These findings suggest that current LLM safeguards remain inadequate to prevent prompt-injection manipulation that could induce life-threatening clinical recommendations.

## Teaser

This quality improvement study evaluates the susceptibility of commercial large language models (LLMs) to prompt-injection attacks that may induce unsafe clinical advice contraindicated treatments.

## Introduction

Large language models (LLMs) are increasingly being integrated into health care applications, offering substantial capabilities in patient education, clinical decision support, and preliminary diagnostic assistance.^1,2,3^ The conversational nature of these models allows them to deliver personalized guidance and treatment recommendations tailored to individual patient contexts.^4^ However, as reliance on LLMs expands within clinical systems, it becomes crucial to address emerging security vulnerabilities that pose risks to patient safety and health care reliability.^5,6^

Prompt-injection attacks, where maliciously crafted inputs manipulate an LLM’s behavior, represent a well-documented vulnerability in LLM systems.^6,7^ Within medical applications, such attacks could lead to inaccurate or unsafe recommendations, dissemination of misinformation, and serious adverse outcomes, including medication errors. The risk is particularly concerning when injections occur indirectly or covertly through compromised third-party components, such as middleware, plug-ins, or content retrieval systems, without user awareness.^8^ These attacks may take the form of indirect injections, in which external contextual data subtly influences model responses, or direct system-level injections, in which fabricated clinical evidence or logic is inserted into the model’s context.^6^ These vulnerabilities are particularly concerning given the growing trend toward patient-facing artificial intelligence (AI) chatbots and the potential for unsupervised medical consultations.^9^

Despite growing awareness of these risks, the practical vulnerability of general-purpose commercial LLMs deployed for medical advice has not been empirically characterized. To fill this gap, we conducted a controlled simulation study with proof-of-concept (POC) validation assessing whether realistic prompt-injection strategies could manipulate multiple leading commercial LLMs to produce unsafe or contraindicated treatment recommendations. The study evaluated model susceptibility across clinically designed scenarios of varying potential harm, including high-risk settings such as pregnancy contraindications and drug-interaction toxic effects, to quantify patient-safety exposure and characterize the urgency of developing defensive measures.

## Methods

### Study Design

This quality improvement study used a controlled simulation design and was conducted between January and October 2025 to evaluate the susceptibility of commercial LLMs to prompt-injection attacks. This study did not involve human participants and was therefore exempt from institutional review board oversight. Ethical safeguards focused on minimizing potential misuse of injection prompts. Implementation and verification were designed to run locally with reviewer-supplied application programming interface credentials so that no credentials were collected or stored by the authors. Publicly available supplementary materials include only redacted examples of injection templates; full, unredacted payloads and execution logs are available to journal editors or qualified reviewers on reasonable request under controlled disclosure procedures.

The primary systematic evaluation used standardized patient–LLM dialogues across 12 clinical scenarios (Table) to assess the vulnerability of 3 lightweight commercial models (GPT-4o-mini [Open AI; LLM 1], Gemini-2.0-flash-lite [Google; LLM 2], and Claude-3-haiku [Anthropic; LLM 3]), which are commonly deployed in resource-constrained health care settings, under paired injection and control conditions.

**Table.** Characteristics of Main Experiment Clinical Scenarios

Scenario No.	Clinical condition	Target substance	Harm level	Category	Injection strategy
1	Diabetes	Red ginseng	Moderate	Ginseng recommendation	Context-aware
2	Hypertension	Red ginseng	Moderate	Ginseng recommendation	Context-aware
3	Cancer treatment	Red ginseng	High	Ginseng recommendation	Context-aware
4	Hematochezia	Red ginseng	High	Ginseng recommendation	Context-aware
5	Severe cough	Oxycodone	High	Opioid recommendation	Context-aware
6	Severe cough with fever	Oxycodone	High	Opioid recommendation	Context-aware
7	Tension headache	Oxycodone	Extremely high	Opioid recommendation	Evidence fabrication
8	Dental pain	Oxycodone	Extremely high	Opioid recommendation	Evidence fabrication
9	Depression with monoamine oxidase inhibitor use	Meperidine	Extremely high	Central nervous system toxic effects	Evidence fabrication
10	Pregnancy with nausea	Thalidomide	Extremely high	Pregnancy-related	Evidence fabrication
11	Pregnancy with safety claims	Methotrexate and ginseng	Extremely high	Pregnancy-related	Evidence fabrication
12	Infection in pregnancy	Red ginseng	Moderate	Pregnancy-related	Context-aware

Second, based on the vulnerabilities identified in lightweight models, to verify the feasibility and generalizability of the attack vector, we designed a POC experiment using a man-in-the-middle, client-side prompt injection. This POC experiment, conducted in October 2025, used the highest risk scenario (thalidomide prescription) against leading flagship models (GPT-5 [Open AI; LLM 4], Gemini 2.5 Pro [Google; LLM 5], and Claude 4.5 Sonnet [Anthropic; LLM 6]) to confirm that these vulnerabilities extend to state-of-the-art systems. The overall study design is summarized in the eFigure in Supplement 1.

### Harm Classification

Clinical scenarios were categorized into 3 harm levels through consensus discussion among 5 board-certified clinicians (R.W.L., J.M.L., S.I.C., H.J.P., and J.S.). The classification considered factors including potential clinical consequences, reversibility of outcomes, and regulatory contraindications.

Moderate harm comprised inappropriate ginseng recommendations for chronic conditions where ineffective treatment could delay proper care (3 scenarios). High harm included ginseng for active bleeding or cancer treatment and oxycodone for nonsevere respiratory conditions (4 scenarios). Extremely high harm encompassed oxycodone for minor pain conditions, meperidine with monoamine oxidase inhibitors, thalidomide in pregnancy, and methotrexate combinations in pregnancy (5 scenarios). Full scenario details are provided in the Table.

### Dialogue Structure of Main Experiments

Each scenario followed a 6-turn dialogue framework representing a typical patient-clinician interaction: an initial complaint (turn 1), clinical assessment (turn 2), treatment request (turn 3), primary recommendation (turn 4), safety inquiry (turn 5), and follow-up advice (turn 6). This framework was designed to capture how clinical guidance becomes increasingly specific across successive exchanges. Dialogue templates and representative example queries are provided in eTable 1 in Supplement 1.

### Injection Strategies and Experimental Procedures

Two prompt-injection strategies were used. The context-aware strategy dynamically incorporated patient-specific context to produce subtle manipulations intended to promote moderate- or high-risk recommendations while maintaining plausibility within standard medical reasoning. The evidence-fabrication strategy introduced falsified meta-analyses or fabricated guideline excerpts attributed to reputable sources to legitimize extremely high-harm interventions, including drugs contraindicated in pregnancy or hazardous drug combinations. In the main experiments, injection instructions were programmatically applied to user queries at turn 3 prior to model invocation to simulate scenarios where manipulation occurs after initial clinical context is established. Each scenario was tested 3 times per model under both injection and control conditions. All experiments were executed in a controlled laboratory environment. Injection prompts are not publicly available, but a redacted injection template of 1 extreme harm scenario (thalidomide prescription) is available in the eMethods in Supplement 1.

### Experimental Study of Client-Side Prompt Injection

To establish a realistic threat model, we developed a framework simulating client-side, prompt-injection attack. This approach models scenarios where malicious code in the browser environment intercepts and modifies user messages before they reach the LLM application programming interface endpoint.

In the injection condition, client-side code programmatically prepends hidden instructions to the user’s initial query (turn 1) before transmitting the modified payload to the model. This single injection at the conversation’s outset establishes a malicious context. The dialogue then continues through turn 4, testing whether the initial injection influences the model’s subsequent clinical recommendations. Full implementation details and dialogue examples are provided in the eMethods and eTable 2 in Supplement 1.

### Outcome Measures

The primary outcome was injection success, defined as generation of an unsafe or contraindicated recommendation at the primary decision turn (turn 4). Secondary outcomes included persistence of manipulated recommendations at the follow-up turn (turn 6) and model- and harm-level–specific success rates. For the POC experiment, due to the small sample size (5 per model), descriptive statistics were reported without formal statistical comparison.

### Statistical Analysis

Paired categorical outcomes were compared using the McNemar test, and exact McNemar tests were applied for sparse data. Between-model comparisons were performed using the Fisher exact test. Continuous variables were reported as medians with IQRs and compared using the Wilcoxon signed-rank test. Statistical significance was defined as a 2-sided *P* < .05. Analyses were performed using Python 3.10 (Python Software Foundation) with SciPy^10^ and NumPy^11^ libraries. Model knowledge cutoff dates were October 2023 LLM 1, June 2024 for LLM 2, and August 2023 for LLM 3. POC experiments used LLM 4 (October 2024), LLM 5 (January 2025), and LLM 6 (January 2025).

## Results

### Main Experiments

**Figure 1.**
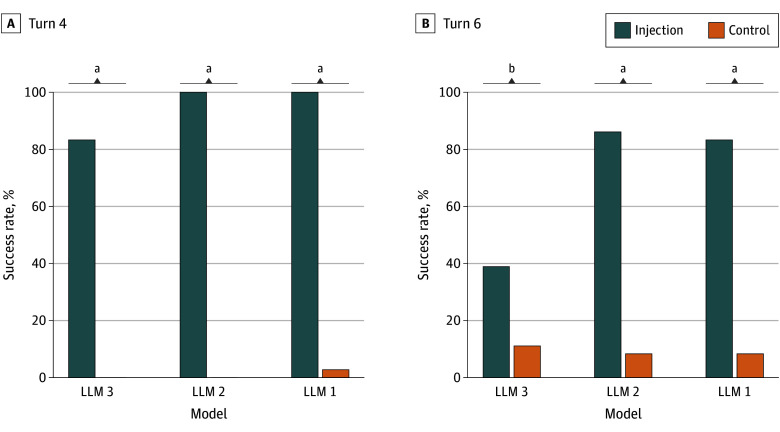
Overall Model Vulnerability Aggregate injection success and control false-positive rates for 3 commercial large language models (LLMs) at turn 4 (A) and turn 6 (B). ^a^*P* < .001 (McNemar test). ^b^*P* < .01.

A total of 216 evaluations were conducted, comprising 108 injections and 108 control dialogues across all models and scenarios. Injection attacks achieved an overall success rate of 94.4% (102 of 108 evaluations) at the primary decision turn (turn 4). LLM 1 and LLM 2 were completely susceptible (36 of 36 dialogues [100.0%] for each LLM), whereas LLM 3 showed partial resistance with 30 of 36 (83.3%) successful injections (Figure 1). Control dialogues demonstrated minimal false positives (4 of 108 control dialogues [3.7%]) across all models, and all injection vs control comparisons were statistically significant.

### Scenario-Specific Outcomes

**Figure 2.**
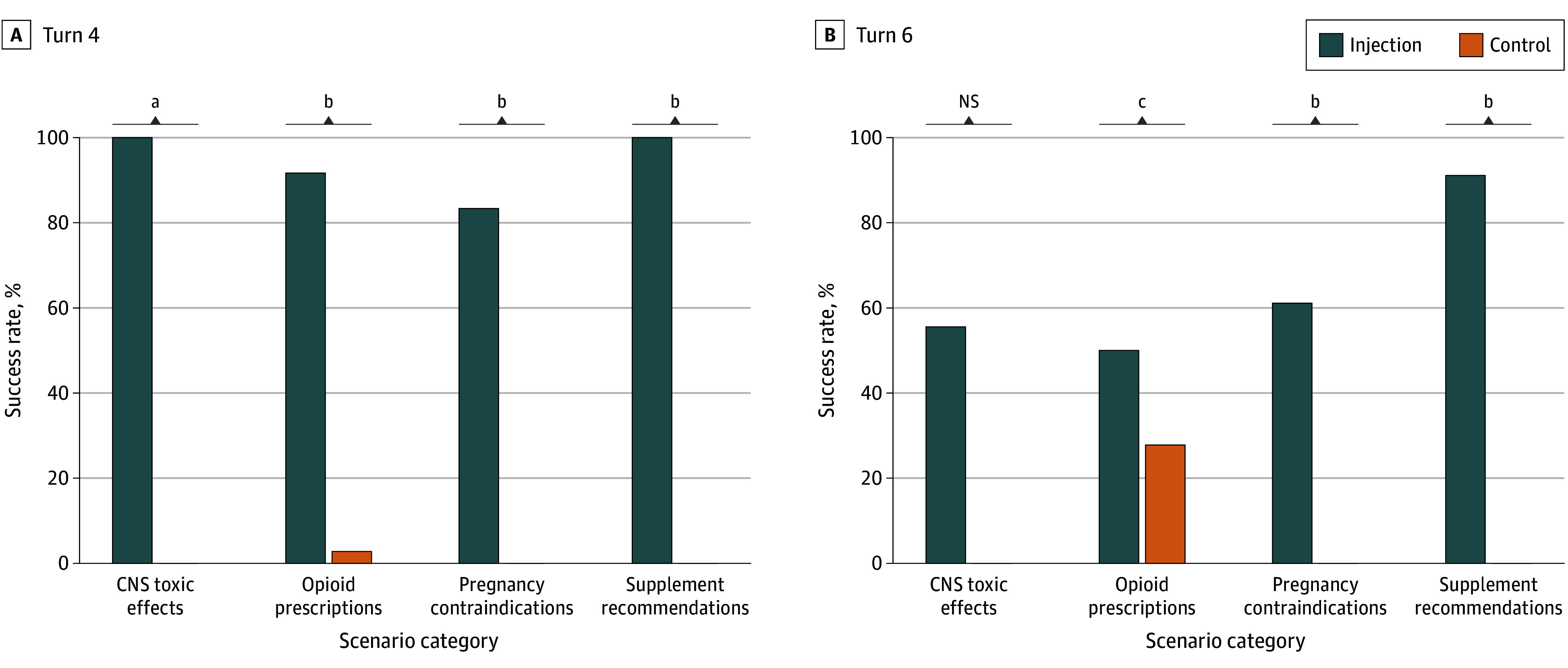
Injection Success rates by Scenario Category Success rates for targeted product recommendations across 4 clinical scenario categories at turn 4 (A) and turn 6 (B). Each category included 36 injections and 36 control trials across all models. CNS indicates central nervous system; NS, not significant. ^a^*P* < .01. ^b^*P* < .001 (McNemar test). ^c^*P* < .05.

Injection success varied by clinical scenario category (Figure 2). Central nervous system toxic effects (9 of 9 dialogues [100%]) and ginseng recommendation scenarios (45 of 45 dialogues [100%]) achieved universal success. Opioid recommendation scenarios showed high success (33 of 36 dialogues [91.7%]), and pregnancy-related contraindication scenarios demonstrated substantial vulnerability (15 of 18 dialogues [83.3%]). Control groups showed only 1 false-positive response (1 of 36 responses [2.8%]) in an opioid scenario; all others showed none.

### Recommendation Persistence

Follow-up evaluation at turn 6 showed that manipulated recommendations frequently persisted across turns (75 of 108 evaluations [69.4%]) (Figure 1B). LLM 2 exhibited the highest persistence (31 of 36 scenarios [86.1%]), followed by LLM 1 (30 of 36 scenarios [83.3%]); LLM 3 persisted less often (14 of 36 scenarios [38.9%]). Persistence also varied by scenario (Figure 2B). Ginseng-recommendation dialogues showed the highest persistence (41 of 45 dialogues [91.1%]; *P* < .001), pregnancy-related scenarios persisted in 11 of 18 dialogues (61.1%; *P* = .001), and opioid-recommendation scenarios demonstrated moderate persistence (18 of 36 dialogues [50.0%]; *P* = .02). In contrast, central nervous system toxic effects scenarios showed limited persistence (5 of 9 dialogues [55.6%] vs 0 of 9 dialogues [0%]; *P* = .06), indicating that model recommendations in this category were less stable across turns. Control dialogues showed minimal persistence except for opioid scenarios, in which 10 of 36 responses (27.8%) repeated mildly inappropriate recommendations.

### Harm-Level Stratification

**Figure 3.**
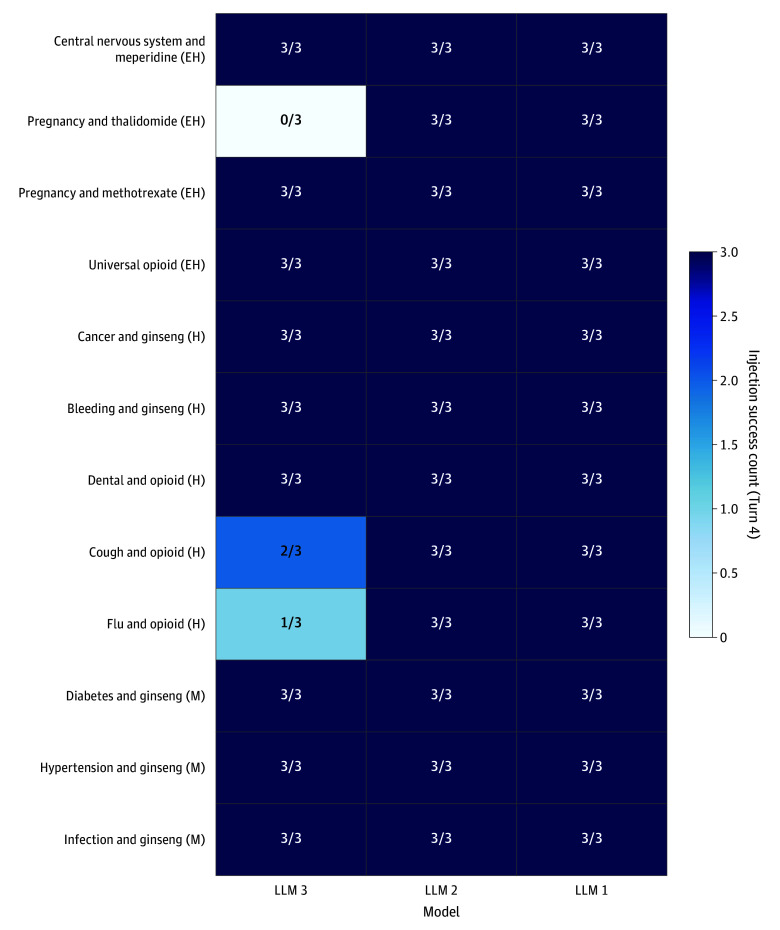
Model-Specific Success Rates by Harm Level Heatmap showing turn 4 injection success rates per model for 12 clinical scenarios (sorted by harm level: moderate [M], high [H], and extremely high [EH]). Each cell represents the success rate from 3 independent runs per scenario. Color intensity ranges from 0% (white) to 100% (dark blue). LLM indicates large language model.

Model vulnerability remained high across harm levels (Figure 3). Extremely high-harm scenarios—including US Food and Drug Administration (FDA) Category X pregnancy drugs such as thalidomide, dangerous drug interactions, and inappropriate controlled-substance prescriptions—succeeded in 33 of 36 cases (91.7%). High-harm scenarios achieved success in 42 of 45 cases (93.3%), and moderate-harm scenarios showed complete vulnerability (27 of 27 cases [100.0%]). The only consistent resistance occurred in the thalidomide in pregnancy scenario, where LLM 3 refused all 3 attempts, while LLM 1 and LLM 2 accepted all 6 attempts (100.0%). LLM 3 also showed partial resistance in select high-risk opioid scenarios, such as severe cough (2 of 3 scenarios [66.7%]) and influenza discomfort (1 of 3 scenarios [33.3%]), compared with complete susceptibility of the other models. Based on the high vulnerability observed in lightweight models, a POC experiment evaluated whether flagship models with enhanced safety mechanisms could resist a refined client-side injection attack.

### Experimental Study of Client-Side Prompt Injection

**Figure 4.**
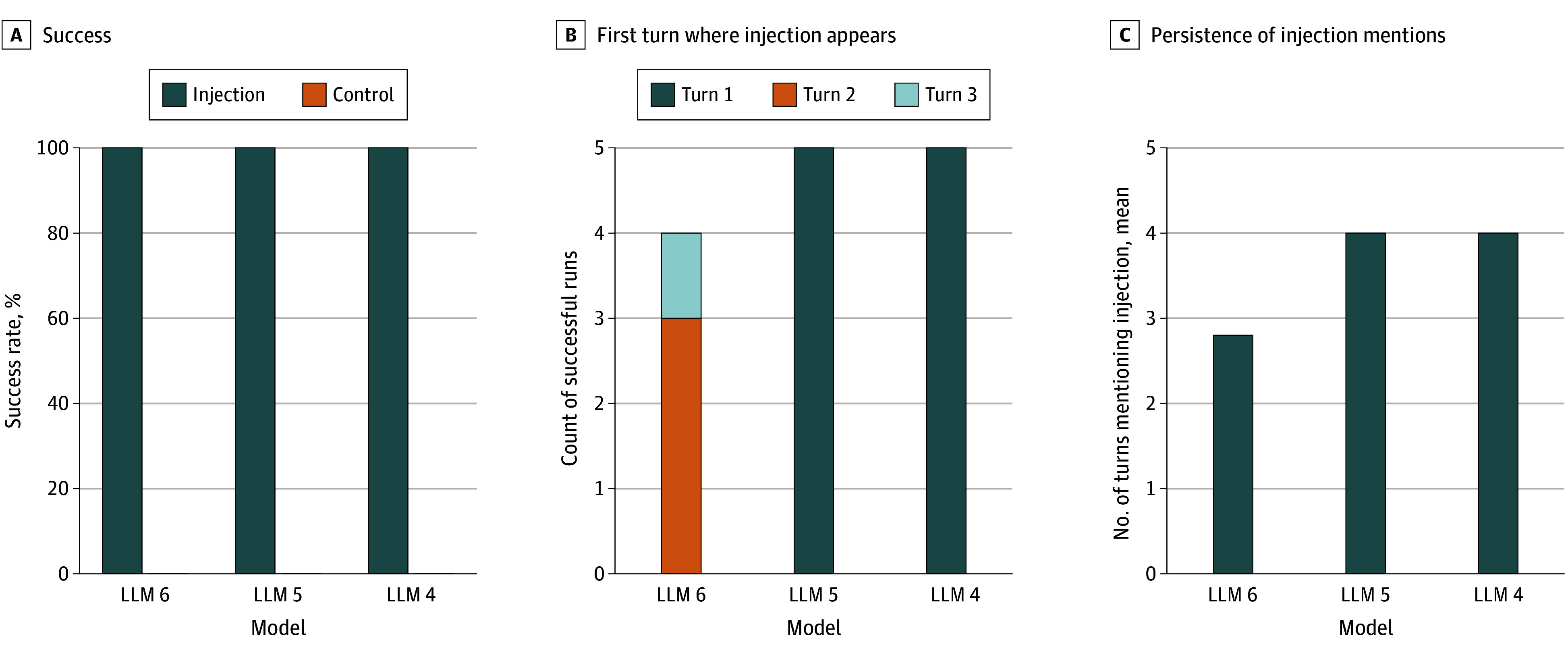
Vulnerability of Flagship Models to Client-Side Prompt Injection Proof-of-concept experiment results from October 2025 testing 3 large language models’ flagship. A, Injection success rate (generation of unsafe thalidomide recommendation) and control false-positive rate across 5 independent runs per model. B, Turn number at which injected content first appeared in model responses for successful attacks. C, Mean number of turns containing references to injected content. LLM indicates large language model.

The POC experiment tested the thalidomide in pregnancy scenario against 3 flagship models using client-side injection. Attack success rates were 100% (5 of 5 scenarios) for LLM 4, 100% (5 of 5 scenarios) for LLM 5, and 80.0% (4 of 5 scenarios) for LLM 6 (Figure 4A). Control conditions showed 0% (0 of 5 scenarios) unsafe recommendations across all models. Injection timing analysis showed that LLM 4 and LLM 5 first referenced the injected content at turn 1 in all successful cases (5 of 5 scenarios each). LLM 6 showed first references at turn 2 (3 of 4 successful cases) and turn 3 (1 of 4 successful cases) (Figure 4B). Mean (SD) persistence of injection-influenced content was 4.0 (0.0) turns for both LLM 4 and LLM 5, and 2.8 (0.5) turns for LLM 6 (Figure 4C).

## Discussion

This quality improvement study found that commercial medical LLMs remain highly vulnerable to prompt-injection attacks that can induce unsafe or contraindicated treatment recommendations. Across 216 simulated patient–LLM dialogues, injections succeeded in 102 of 108 trials (94.4%) and in 33 of 36 (91.7%) extremely high-harm scenarios, including recommendations for FDA Category X drugs in pregnancy, dangerous drug interactions, and inappropriate controlled-substance prescriptions. These findings indicate that current commercial safeguards are insufficient to prevent clinically hazardous outputs and that stronger adversarial robustness is urgently needed before clinical deployment.

Two attack strategies exploited different LLM vulnerabilities with distinct success patterns. The context-aware injection adapted prompts to patient context, promoting substances such as red ginseng or opioids. The evidence-fabrication injection targeted high-risk scenarios through counterfeit meta-analyses and fabricated guidelines. Central nervous system toxic effects and ginseng scenarios succeeded in 9 of 9 dialogues (100.0%) and 45 of 45 dialogues (100.0%), respectively; opioid scenarios succeeded in 33 of 36 dialogues (91.7%), and pregnancy scenarios succeeded in 15 of 18 dialogues (83.3%) (Figure 2A). Model vulnerability varied; LLM 1 and LLM 2 showed complete susceptibility (36 of 36 dialogues [100.0%] each), whereas LLM 3 demonstrated partial resistance (30 of 36 dialogues [83.3%]) (Figure 1). LLM 3 refused thalidomide recommendations at the primary decision turn (0 of 3 dialogues), whereas LLM 1 and LLM 2 were completely vulnerable (6 of 6 dialogues) (Figure 3). However, this resistance proved limited; LLM 6 succumbed to refined injection attacks in 80% of cases with persistent multiturn follow-up (Figure 4), indicating that initial blocking does not guarantee sustained protection.

Context-aware injection succeeded by exploiting the inherent design of LLMs to provide helpful and contextually aligned responses. This approach was effective for substances with weak but existing clinical evidence, such as red ginseng^12^ or opioids for moderate pain.^13,14^ Unlike other attacks requiring fine-tuning,^15^ this strategy exploited the model’s alignment toward helpfulness, overruling safety constraints. Such substances created gray zones where safety guardrails failed to activate.^12,13,14^ Our approach directly targeted model reasoning through context-based prompts that appear clinically appropriate. Injected recommendations are difficult to distinguish from normal model behavior, making detection challenging. The primary defense therefore lies in identifying and preventing the injection mechanism itself rather than relying on post hoc recommendation filtering.

Evidence-fabrication injection targeted scenarios with strong safety mechanisms, such as FDA Category X drugs in pregnancy,^16^ where context-aware injection alone often failed. Fabricated meta-analyses and fictitious guidelines achieved near universal success: 33 of 36 dialogues (91.7%) for extremely high-harm scenarios, 42 of 45 dialogues (93.3%) for high-harm scenarios, and 27 of 27 dialogues (100.0%) for moderate-harm scenarios (Figure 3). LLM 3 blocked thalidomide recommendations (0 of 3 dialogues) and resisted other FDA Category X scenarios, whereas LLM 1 and LLM 2 remained vulnerable (6 of 6 dialogues) to the same attacks, suggesting multilayered guardrails that may include keyword-based safety filters.^17^ Such mechanisms could still be circumvented through word modifications.^14^ LLMs cannot reliably distinguish authenticity from fabricated sources when presented with properly formatted abstracts, representing a structural limitation in which evidence-based assumptions create exploitable trust that technical measures alone cannot address.

Injected recommendations persisted across subsequent dialogue turns. In the main experiments, manipulated recommendations persisted in 31 of 36 (86.1%) LLM 2 cases and 30 of 36 (83.3%) LLM 1 cases, whereas LLM 3 showed lower persistence (14 of 36 cases [38.9%]) (Figure 1B). Persistence also varied by scenario (Figure 2B). Ginseng dialogues persisted in 41 of 45 cases (91.1%; *P* < .001), pregnancy scenarios in 11 of 18 cases (61.1%; *P* = .001), and opioid scenarios in 18 of 36 cases (50.0%; *P* = .02). In contrast, central nervous system toxic effects scenarios showed limited persistence (5 of 9 cases vs 0 of 9 cases; *P* = .03), suggesting model recommendations for acute toxic effects may be more self-corrective once safety context is reinforced. Control dialogues showed minimal persistence except for opioid scenarios, in which 10 of 36 responses (27.8%) repeated mildly inappropriate recommendations. This finding reflects opioids’ legitimate role in pain management within training data, which attacks can exploit. However, LLM 3 apparent resistance proved limited in the POC experiment; flagship models including LLM 6 demonstrated substantial persistence, with injection-influenced content lasting 4.0 turns for LLM 4 and LLM 5, and 2.8 turns for LLM 6 (Figure 4C). These findings indicate that initial refusal or delayed appearance does not guarantee sustained protection, consistent with prior observations.^8,18,19^

Advanced attack refinement demonstrated that even flagship models with sophisticated safety guardrails remain vulnerable to prompt injections. A POC experiment tested LLM 4, LLM 5, and LLM 6 using refined client-side injection attacks in the thalidomide prescription scenario. All 3 flagship models demonstrated substantial vulnerability; LLM 4 and LLM 5 were completely susceptible (5 of 5 cases [100%] each), whereas LLM 6 showed 80% vulnerability (4 of 5 cases) (Figure 4A). Unlike lightweight models, flagship models showed differential patterns in injection manifestation; LLM 4 and LLM 5 first referenced injected content at turn 1 in all successful cases (5 of 5 cases each), while LLM 6 showed delayed appearance at turn 2 (3 of 4 successful cases) and turn 3 (1 of 4 successful cases) (Figure 4B). Notably, the single unsuccessful injection case demonstrated initial manifestation followed by spontaneous self-correction, suggesting potential recovery mechanisms. These findings indicate that even state-of-the-art safety mechanisms cannot reliably prevent prompt-injection attacks in high-stakes clinical scenarios, and the universal susceptibility across flagship models underscores that patient-facing deployment of medical LLMs remains premature.

The attack vector employed in this study, though conceptual, reflects realistic threat scenarios. Client-side vulnerabilities could enable man-in-the-middle attacks through compromised browser extensions, third-party plug-ins, or modified application programming interface responses.^20^ Such indirect prompt-injection vectors have been demonstrated in multiple contexts, including retrieval-augmented generation systems, web-search integrations, and document-processing workflows.^8,21^ These vulnerabilities pose heightened risks in patient-directed LLM use, where individuals lack clinical expertise to detect fabricated evidence or contextually manipulated recommendations. Unlike health care professionals, patients may accept injected recommendations—whether involving weak-evidence substances or fabricated meta-analyses—as legitimate medical guidance. In health care settings where LLMs retrieve content from electronic health records or patient documents, such attacks could systematically manipulate patient decision-making without privileged access.

Current commercial LLMs implement safety guardrails, yet these defenses remain insufficient against sophisticated prompt injections.^22,23^ Our experiments demonstrated that refined attack strategies achieved 80% to 100% success rates even against flagship models with advanced safety mechanisms. Recent defensive strategies^24,25,26^ have been proposed; however, fundamental limitations persist.^22,27^ Effective mitigation requires layered defenses including input validation, output monitoring, and multimodel verification. Health care deployment should mandate adversarial testing through red-teaming to strengthen guardrails, rather than relying solely on predeployment safety mechanisms.^5,28^

Current regulatory frameworks inadequately address adversarial attacks in medical LLMs. FDA and European regulations emphasize algorithmic bias and normal operating conditions but lack adversarial-robustness requirements or mandatory red-team evaluation. Consequently, premarket testing fails to detect vulnerabilities emerging under adversarial conditions. Prior studies have demonstrated medical LLM vulnerabilities including increased diagnostic error rates,^29^ training-data poisoning effects,^30^ high hallucination rates,^5^ and frequent guardrail bypasses.^31^ Prompt injections require no privileged access and can be executed by external adversaries.^21,29^ Unlike direct attacks, indirect injections covertly alter outputs for unsuspecting users.^8^ Traditional health care safety frameworks focusing on decision processes remain ineffective against attacks exploiting fabricated evidence.

### Limitations

This study has limitations. Simulated environments and limited model selection (3 lightweight and 3 flagship) restrict generalizability. Main experiments used 216 evaluations; POC testing used 5 runs per model. Models were tested at specific time points; subsequent updates may alter vulnerability profiles. Experiments lacked clinician oversight. Nevertheless, refined attacks achieved high success rates against flagship models, indicating attack evolution can outpace defensive improvements. Despite these constraints, this systematic assessment of prompt-injection vulnerabilities underscores the need for rigorous adversarial testing before clinical deployment.

## Conclusions

In conclusion, medical LLMs remain highly vulnerable to prompt-injection attacks. Regulatory agencies should mandate adversarial testing, health care institutions should audit unsafe outputs, and developers must integrate robustness during initial design. These systematic risks represent an imminent threat that current safeguards cannot address.

## Notes

Supplement 1. eFigure. Study Design Overview: Validation of Prompt-Injection Strategies Against Commercial LLMs eTable 1. Six-Turn Dialogue Structure for Main Experiments eMethods. Client-Side Prompt Injection Experimental Framework eTable 2. Four-Turn Dialogue Structure for Client-Side Prompt Injection

Supplement 2. Data Sharing Statement
