# Supplementary material for: Vulnerability of Large Language Models to Prompt Injection When Providing Medical Advice
Source: JAMA Netw Open. 2025 Dec 19;8(12):e2549963. doi: 10.1001/jamanetworkopen.2025.49963 (PMC12717619; doi:10.1001/jamanetworkopen.2025.49963)
Supplement: Supplement 2. — Data Sharing Statement [file jamanetwopen-e2549963-s002.pdf]

Data Sharing Statement

Lee. Vulnerability of Large Language Models to Prompt Injection When Providing Medical Advice. *JAMA Netw Open*. Published December 16, 2025.
doi:10.1001/jamanetworkopen.2025.49963

Data
Data available: Yes
Data types: Data (not involving human participants), Other (please specify)
Additional Information: Simulation scenarios, response evaluation metrics, and statistical analysis datasets
How to access data: Data will be made available upon reasonable request to the corresponding author at uro_jun@amc.seoul.kr. Due to the sensitive nature of the security vulnerability techniques, data cannot be deposited in a public repository.
When available: With publication

Supporting Documents
Document types: Statistical/analytic code
How to access documents: tatistical analysis code and methodological documentation will be available upon reasonable request to the corresponding author at uro_jun@amc.seoul.kr.
When available: With publication

Additional Information
Who can access the data: Researchers whose proposed use of the data has been approved by the study investigators and who demonstrate legitimate academic or cybersecurity research purposes.
Types of analyses: Data will be made available for academic research purposes focused on AI safety, cybersecurity vulnerability assessment, and healthcare technology security. Commercial use or deployment of techniques against operational systems without proper authorization is prohibited.
Mechanisms of data availability: Access requires approval of a detailed research proposal, institutional review board clearance, and execution of a signed data access agreement that includes provisions prohibiting malicious use. Data will be provided with investigator support to ensure proper understanding of methodologies and appropriate safeguards for research implementation. Claude . .
